# Worldwide prevalence of the double burden of malnutrition in children and adolescents at the individual level: systematic review and meta-regression

**DOI:** 10.1016/j.jped.2024.11.010

**Published:** 2025-01-31

**Authors:** Raytta Silva Viana, Keisyanne De Araújo-Moura, Augusto César Ferreira De Moraes

**Affiliations:** a Universidade de São Paulo, Faculdade de Saúde Pública, Departamento de Epidemiologia, Programa de Pós-Graduação em Saúde Pública, São Paulo, São Paulo, Brazil; b Universidade de São Paulo, Faculdade de Medicina, Grupo de Pesquisa YCARE Research Group (Youth/Child Cardiovascular Risk Environmental Research Group), São Paulo, São Paulo, Brazil; c Universidade de São Paulo, Faculdade de Saúde Pública, Programa de Pós-Graduação em Saúde Pública, Departamento de Saúde Pública, São Paulo, São Paulo, Brazil; d The University of Texas Health Science Center at Houston, School of Public Health in Austin, Department of Epidemiology, Michael & Susan Dell Center for Healthy Living, Texas Physical Activity Research Collaborative (Texas PARC), Austin, TX, USA

**Keywords:** Obesity, Overweight, Nutrition disorder, Malnutrition, Undernutrition, Micronutrient deficiency

## Abstract

**Objective:**

This study aimed to assess the prevalence of the double burden of malnutrition (DBM) at the individual level in children and adolescents through a comprehensive literature review.

**Sources:**

Electronic databases, including PubMed, Scopus, and Web of Science, were searched for articles published up until September 9, 2022. Studies reporting individual-level DBM in children and adolescents were included, and meta-regression models were used to investigate potential causes of heterogeneity across studies.

**Summary of the findings:**

Of the 784 articles initially retrieved, 11 met the inclusion criteria. The overweight/obesity prevalence ranges from 8.1 % to 37.0 %, and the undernutrition (stunting, micronutrient deficiency, or anemia) from 4.2 % and 73.0 %. The prevalence of DBM ranged from 1 % to 35.4 %, with the highest rates observed in low- and middle-income countries. Among children, Asia reported the highest DBM prevalence, while in adolescents, Latin America had the highest rates. The review revealed significant variability in DBM prevalence across studies, with a notable increase in research on this topic over the past decade (2013–2022).

**Conclusion:**

These findings underscore the concerning global prevalence of the double burden of malnutrition in children and adolescents, particularly in low- and middle-income countries. Standardized definitions and methods are urgently needed to improve comparability, along with further research to identify the specific drivers of DBM and inform effective prevention strategies. CRD42022333424.

## Introduction

Obesity is a chronic non-communicable disease (NCD) that affects various systems in the body and is a significant risk factor for several other NCDs, including type 2 diabetes, cardiovascular disease, hypertension, stroke, different forms of cancer, and mental health issues.^1^ Currently, around the world, approximately 340 million adolescents and 39 million children are overweight, and it is projected that by 2025, about 167 million people globally will experience compromised health due to being overweight or obese.^2^

Undernutrition continues to be a significant problem that manifests in different forms, such as stunting, underweight, and micronutrient deficiencies, with serious short- and long-term consequences, including increased morbidity and mortality, irreversible physical and neurocognitive damage, and an increased risk of developing NCDs in adulthood.^3^ Currently, 33 countries have at least 30 % of children who are stunted worldwide, with an estimated 151 million children under the age of 5 being affected, 47 million underweight for their age, and 340 million micronutrients deficient. Undernutrition is responsible for 45 % of child deaths globally.^2^

The Double Burden of Malnutrition (DBM) is the simultaneous occurrence of undernutrition and overweight/obesity and has gained more attention recently, as it appears to be more persistent and widespread than previously thought.^4^ One in three Low and Middle-Income Countries (LMICs) is affected by undernutrition and obesity globally. In the 2010s, 38 % of the 126 LMICs faced DBM, with a particularly high prevalence in sub-Saharan Africa, South Asia, East Asia, and the Pacific. DBM is increasing at a rate that is 30 % faster in children in developing countries compared to high-income countries.^5^

DBM can occur at various levels, including the national level where both overweight and undernutrition coexist in the same population, the family level or mother-child pairs where the mother may be overweight and one of her children under five is wasted, or the mother is overweight and one of her children under five is stunted, or the mother is thin and one of her children is overweight. It can also occur at the intra-individual level where an individual has both excess weight and micronutrient deficiency. These different levels of DBM highlight the complexity of the issue and the need for a multi-faceted approach to address it.^4^, ^5^, ^6^

Exposure to DBM for an extended period increases the risk of developing a range of health problems, such as cardiovascular diseases and deficiencies in cognitive development.^7^ This condition in children and adolescents deserves special attention, as the coexistence of undernutrition and obesity can exacerbate the vulnerability of this age group to chronic diseases in the future.^6^^,^^8^ Furthermore, understanding DBM from childhood is essential to inform more effective public policies, as the early years of life are critical for the development of healthy metabolic and immune systems.^9^ Therefore, DBM at the individual level, especially in children and adolescents, represents a global public health issue, requiring long-term interventions to reduce both undernutrition and obesity rates.^10^

However, there is still a lack of comprehensive assessment of the prevalence of DBM at the individual level in the child population across different countries. The absence of data on this condition at the individual level in childhood prevents health policies from being effectively adapted to combat multiple forms of undernutrition simultaneously.^10^, ^11^, ^12^, ^13^, ^14^ Moreover, focusing on children and adolescents is crucial, as this age group is in a critical period for physical and cognitive development, and appropriate interventions can prevent progression to more severe chronic conditions in adulthood. To assess the prevalence of DBM in children and adolescents at an individual level, this study proposes conducting a systematic review of the current literature.

## Methods

This systematic review utilized the methodology proposed by Clark & Oxman,^15^ focusing on studies reporting the prevalence of DBM in children and adolescents aged 2–19 years.

### Identification of eligible studies—Electronic search and other sources

The authors conducted searches in three electronic databases: Medline/PubMed, Scopus, and Web of Science, with searches up to September 9, 2022. This review was registered in the PROSPERO International Prospective Register for systematic reviews on January 12, 2022, under reference number CRD42022333424. A comprehensive search was conducted using specific search terms, subject title truncations (*), and Boolean operators ("AND," "OR"), tailored to each database's requirements.

Search strategy groups included:

i. The first group included terms related to the study population: child, children, childhood, school, preschool, preschoolers, child preschool, adolescents, teen, teenager, youth, and adolescence;
ii. The second group included terms related to excess weight and micronutrient deficiencies: obesity, metabolic diseases, overnutrition, overweight, body weight, pediatric obesity, iron metabolism disorders, iron deficiencies, iron, anemia, iron deficiency, and iron deficiency; The search terms focused on iron deficiency and overweight were selected due to the high prevalence of iron deficiency as a micronutrient deficiency in children and adolescents, as well as its significant association with overweight/obesity. This specificity aligns with existing literature on the DBM, where iron deficiency is frequently reported as a key component.^16^^,^^17^
iii. The third group added terms related to DBM: nutritional deficiency, nutritional deficiencies, undernutrition, malnourishment, malnourishment, nutrition disorders, the double burden of malnutrition, and malnutrition;
iv. As the aim of this review was to determine the prevalence of DBM, a fourth set of commands was added to restrict the study design: prevalence studies, prevalence, cross-sectional studies, and surveys.

**Figure 1:**
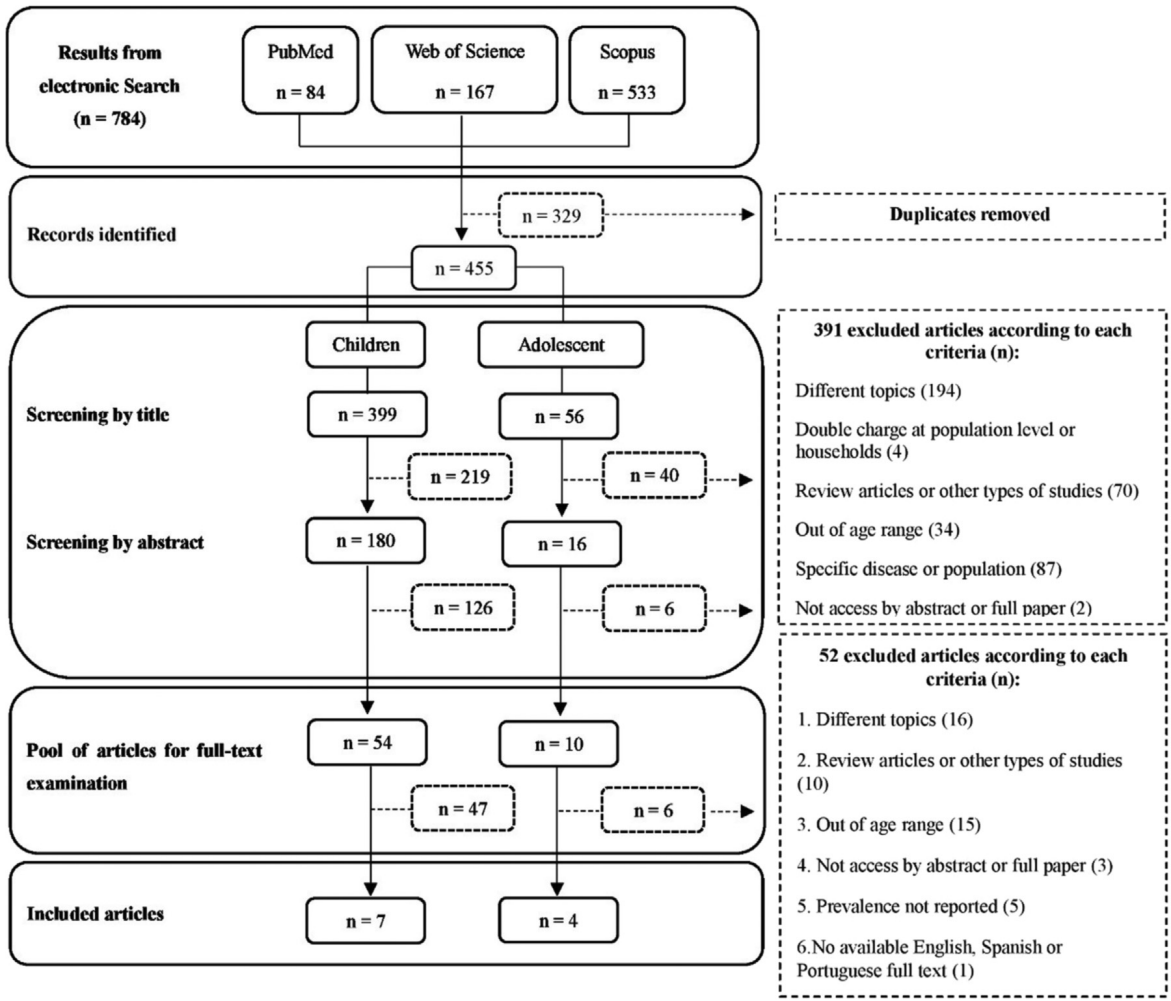
Selection process and the number of articles included in the review.

The search strategy was executed initially in September 2022 and rerun in December 2022 before the final analysis. A flow diagram (Figure 1) illustrates the selection process and number of records retrieved.

The following inclusion and exclusion criteria were applied to identify eligible studies:

*Inclusion criteria:*

i. Original research articles;
ii. Studies reporting the prevalence of double burden of malnutrition using the individual criterion where the same individual is overweight and micronutrient deficient concurrently;
iii. Studies conducted on children and adolescent populations aged between 2 and 19 years;
iv. Studies published up until the end of September 2022;
v. Full-text studies are available in English, Spanish, or Portuguese.

*Exclusion criteria:*

i. Review articles, including meta-analyses, were excluded;
ii. Studies that focused on specific diseases or specific populations (such as refugees) were excluded;
iii. Studies that reported only on the prevalence of double burden of malnutrition at the household level, rather than at the individual level, were excluded.

### Assessment, data extraction, and analysis

This study followed the systematic review methodology as proposed in the Preferred Reporting Items for Systematic Reviews and Meta-Analyses (PRISMA) guidelines. The PRISMA 2020 statement includes a checklist of 27 essential items that ensure transparent reporting.^18^

The studies retrieved from various databases were imported into EndNote, which is a bibliographic reference manager developed by Clarivate Analytics, and duplicates were removed. In cases where the title and abstract of each study contained insufficient information to determine its suitability for inclusion, the complete manuscript was reviewed. If there were discrepancies in the inclusion of studies between the two authors, a third author was involved in the evaluation. After screening the records in the search results by titles and abstracts, the authors examined the full-text versions of all identified articles to determine their potential eligibility. A research log was maintained for accountability and transparency.

*The following items were included in the database:*

i. Publication information: Name of the first author, name of the journal, year of publication.
ii. Data: Country, year of data collection, and number of subjects analyzed.
iii. Methods: Focus on the combination of people with malnutrition and overweight at the same time, age group of children and adolescents, and nutritional indicators used to identify malnutrition and overweight.
iv. Results: Number and prevalence of individuals with a double burden of malnutrition, along with associated factors.

For comparison purposes, crude prevalence rates were preferably retrieved, if available, as some studies reported only raw (unadjusted) values. In studies that analyzed multiple datasets (multiple years) or that used multiple indicators, information was extracted for each outcome.

### Summary of results

The extracted prevalence estimates were exported to Statistical and Data Science Software [(STATA)/SE V.15.1] for subsequent meta-regression analysis. The data were synthesized based on nutritional status (overweight/obesity and nutritional deficiency). The authors estimated the between-study variance (tau2), the residual variation attributable to statistical heterogeneity (I2), the proportion of between-study variance explained (R2), and the joint test for all covariates. A *p*-value ≤ 0.05 was considered significant in the meta-regression. The potential cause of heterogeneity among factors that may determine the prevalence of DBM was explored through meta-regression analysis.^19^ Results were presented in narratives, tables, and all statistical interpretation was reported based on a *p*-value < 0.05 and 95 % CI.

## Results

### Study selection and data collection

The literature search resulted in a total of 784 article titles, of which 64 full-text articles were screened. Finally, 11 articles were deemed eligible for inclusion in the study based on the established criteria. Table 1 provides a summary of the selected articles, including information on the main author, country of the study, year of publication, total number of participants, age group, and proportion of girls. All of the studies were published after 2008, although the concept of DBM has been around for more than 20 years. The earliest publication found in this study that reported the prevalence of DBM at the individual level in children and adolescents was published in 2009.

**Table 1:** Descriptive analysis of the reviewed studies.

Authors	Country	Year published	*n* total study	Age range	Proportion of girls (%)
Ortega et al.^20^	Venezuela	2009	74	14–19 y	100
Iriart et al.^30^	EUA	2013	14,710	2–19 y	48.6
Kordas et al.^21^	Colombia	2013	655	13–17 y	100
Kroker-Lobos et al.^22^	Mexico	2014	16,351	5–11y	49.8
Piernas et al.^25^	China	2015	2839	2–12 y	46.3
Zou et al.^26^	China	2016	1534	7–17 y	49.4
Jones et al.^23^	Mexico	2017	4039	15–19 y	100
Crivelli et al.^27^	Tajakistan	2018	1342	2–5y	48.7
Hoang et al.^28^	Vietnam	2019	893	6–9 y	50.4
Zarate-Ortiz et al.^24^	Mexico	2019	7380	12–19 y	50.6
Moyo et al.^29^	South Africa	2022	237	6–18 y	50.2

The 11 articles included in this review reported data from 8 countries, with most studies conducted in low- and middle-income countries. Five studies were conducted in Latin America (*n* = 5),^20^, ^21^, ^22^, ^23^, ^24^ four in Asia (*n* = 4),^25^, ^26^, ^27^, ^28^ one in Africa (*n* = 1),^29^ and one in North America (*n* = 1).^30^ The synthesis included descriptive information obtained from 50,054 individuals between the ages of 2 and 19 years. Of the included articles, seven^21^, ^22^, ^23^, ^24^, ^25^, ^26^, ^27^ used secondary data such as Demographic and Health Surveys. Some studies focused on specific populations or contexts within the country, such as Hispanics (*n* = 1)^30^ rural areas (*n* = 2)^26^^,^^28^ and women of reproductive age (*n* = 3).^21^^,^^23^^,^^29^ Only one^26^ study reported multiyear prevalence rates for the study country, and the age range of children and adolescents varied between studies, with one^27^ study stratifying age in months.

### Cutoff points

For the classification of overweight and obesity, Body Mass Index (BMI) for age was the commonly used indicator for nutritional status classification. Most studies (*n* = 9)^20^, ^21^, ^22^, ^23^, ^24^^,^^27^, ^28^, ^29^, ^30^ used the BMI for age z-score, or Body Mass Index for Age (BAZ), where BAZ > +1 Standard Deviation (SD) to define overweight, including obesity. However, two studies^25^^,^^26^ used the BMI classification for those over 19 years old, with one^25^ using BMI > 25.0 kg/m² and the other^26^ using BMI > 24.0 kg/m² for overweight. Among studies that used z-scores (according to Child Growth Standards), the World Health Organization (WHO) references published in 2006^31^ for children under 5 years of age (BAZ > +1SD as "at risk of overweight," BAZ > +2SD as overweight, and BAZ > +3SD as obese) and in 2007^32^ for those aged 5 to 19 years (BAZ > +1SD for overweight and BAZ > +2SD for obesity) were most commonly used for classification.

For nutrient deficiencies, the studies considered different micronutrients and/or growth deficits. The most commonly evaluated micronutrient was iron, and the studies used the following cutoff points for iron deficiency: Ferritin = 15–20 µg/L and Ferritin < 12.0 mg/L. Additionally, some studies showed deficiencies in specific micronutrients such as iron, iodine, vitamin D, vitamin B12, and folate. Only one study measured vitamin D and iodine, and another study measured vitamin B12 and folate, while nine studies analyzed anemia and four studies analyzed growth retardation. For the classification of anemia, the most commonly used marker was Hemoglobin (Hb) with the cutoff point established by the WHO, where Hb < 120 g/L.^33^ For growth retardation, the cutoff values were Height for Age (HAZ) < −2 SD, according to the WHO references published in 2006 for children under 5 years of age and in 2007 for those aged 5 to 19 years.^31^^,^^32^

### DBM prevalence

Table 2 presents the prevalence rates of overweight and obesity, malnutrition, developmental delays, and micronutrient deficiencies for each survey analyzed in this study. The table includes the specific criteria used to classify each condition, as well as the number of prevalence values reported in the included articles. A total of 23 prevalence rates were analyzed, as six studies used multiple criteria for classifying developmental delays, and two studies were conducted in different years and stratified into different age groups.

**Table 2:** Description of the prevalence of overweight, micronutrient deficiencies, DBM, established criteria and cutoff points for classifying overweight, micronutrient deficiencies, and definition of the DBM.

Authors	Classification of weight status	WOB (%)	Classification of undernutrition	Undernutrition (%)	DBM (%)
Ortega et al.^20^	BMI > percentile 90, Venezuela Project	8.1	Anemia -> Hb < 120 g/l, WHO and INACG; ID - > Ferritin = 15–20 ug/l, WHO and CESNI	Anemia = 48.6; DI = 13.5	Anemia + WOB = 1.3 DI + WOB = 1.35 Anemia + DI + WOB = 5.4
Iriart et al.^30^	BMI ≥ percentile 85, CDC	31.9	Stunting -> HAZ < percentile 5; VitD; DI; Iodine, CDC	Stunting = 3.5; VitD = 5.6; Iodine = 22.1; Iron = 5.3	Stunting + WOB = 2.5 VitD + WOB = 8.0 Iodine + WOB = 19.4 Iron +WOB = 6.4
Kordas et al.^21^	BAZ ≥ 1 SD, WHO	20.2	Anemia -> Hb < 120 g/l; ID -> Ferritin <12,0 mg/l, CIFW	Anemia = 32.0; ID = 12.5	Anemia + WOB = 7.6 DI + WOB = 2.1
Kroker-Lobos et al.^22^	BAZ ≥ 1 SD, WHO	34.4	Anemia -> Hb < 120 g/l, WHO; Stunting -> HAZ, WHO	Anemia = 10.1; Stunting = 6.9	Anemia + WOB = 2.9 Stunting + WOB = 1,0
Piernas et al.^25^	BMI ≥ 25, IOTF	a2009: 2–6y = 14.3; 7–12y = 14.2 2011: 2–6y = 22.5; 7–12y = 18.9	Stunting -> HAZ, WHO	Stunting 2009: 2–6y = 4.5; 7–12y = 2.4 2011: 2–6y = 4.2; 7–12y = 0.4	Stunting + WOB 2009: 2–6y = 2.7; 7–12y = 0.5 2011: 2–6y = 3.2; 7–12y = 0.4
Zou et al.^26^	BMI ≥ 24, GCOTF	19.8	Anemia -> Hb <115 g/l (5–11 y) Hb <120 g/l (12–14 y and girls > 15y) Hb <130 g/l (boys > 15y), WHO and UNICEF	Anemia = 5.2	Anemia + WOB = 6.1
Jones et al.^23^	BAZ ≥ 1 SD, WHO	37.0	Anemia -> Hb < 120 g/l, WHO	Anemia = 7.4	Anemia + WOB = 1.8
Crivelli et al.^27^	BMI ≥ percentile 85, WHO	22.5	Anemia -> Hb < 11 g/dll, WHO and UINICEF	Anemia = 20.0	Anemia + WOB = 33.3
Hoang et al.^28^	BAZ ≥ 1 SD, WHO	18.7	Anemia -> Hb < 115 g/l, WHO	Anemia = 12.9	Anemia + WOB = 7.9
Zarate-Ortiz et al.^24^	BAZ ≥ 1 SD, WHO	30.9	Anemia -> Hb <120 g/ (12 −14 y and girls > 15y) Hb <130 g/l (boys > 15y), WHO; Stunting -> HAZ, WHO	Anemia = 8.5; Stunting = 16.8	Anemia + WOB = 35.4 Stunting + WOB = 25.1
Moyo et al.^29^	BAZ ≥ 1 SD, WHO	18.2	Anemia -> Hb < 11.8 g/dl (6–11 y) Hb < 12.6 g/dl (12–15 y boys) Hb < 11.9 g/dl (12–15y girls) Hb 13.6 g/dl (16–19y boys) Hb 12.0 g/dl (16–19y girls), CDC; VitB12 < 156 pmol/, D-A=CHl; Folato < 5.9 nmol/dl, WHO	Anemia = 6.3; VitB12 = 4.2; Folate = 73.0	Folate + WOB = 13.1 one or more UN + WOB = 13.6

WOB, Overweight/Obesity;UN, Undernutrition; BAZ, BMI z-score; HAZ, height-for-age z-score; Hb, Hemoglobin; ID, Iron Deficiency; VitD, Vitamin D; WHO, World Health Organization; IOTF, International Task Force against Obesity; GCOTF, Group of China Obesity Task Force; INACG, International Advisory Group of anemia; CESNI, Associated Center of the Faculty of Medicine of the Salvador's university; UNICEF, United Nations Children's Fund; CDC, Center for Disease Control and Prevention; D-A-CHI, Nutrition societies of Germany, Austria and Switzerland; y, years.

a Multi-year study (2009 and 2011).

The highest prevalence of DBM among children was found in Asia,^27^ while in adolescents, it was found in Latin America (24). Over 90 % of the articles^21^, ^22^, ^23^, ^24^, ^25^, ^26^, ^27^, ^28^, ^29^, ^30^ reported a prevalence greater than 14 % for overweight and obesity. The prevalence of stunting,^24^ micronutrient deficiency,^20^^,^^21^^,^^27^^,^^29^^,^^30^ or anemia^20^, ^21^, ^22^^,^^27^^,^^28^ was equal to or greater than 10 % in the presented studies. The prevalence of anemia and micronutrient deficiencies, including iron, iodine, vitamin D, vitamin B12, and folate, were reported in the same research in three studies.^20^^,^^21^^,^^29^ The prevalence of growth deficit and anemia was reported in three studies,^22^^,^^24^^,^^29^ while the prevalence of growth deficit and micronutrient deficiency was reported in one study.^16^ Iron was the most frequently studied micronutrient deficiency.^20^^,^^21^^,^^30^

### Meta-regression of factors that may determine the prevalence of DBM

A meta-regression analysis was conducted to identify the potential sources of heterogeneity in factors that may determine the prevalence of DBM, due to the presence of variability in these factors. The analysis considered the year of publication of the studies, sample size, age range of individuals, economic classification of countries,^34^ classification used for nutritional status, prevalence of overweight/obesity and nutritional deficiency. However, none of the variables included showed a statistically significant source of heterogeneity in all analyses, as shown in Table 3.

**Table 3:** Meta-regression to identify heterogeneity between the factors that can determine the prevalence of DBM in children and adolescents.

Variables	Coefficient	*P* < 0.05	95 % CI
Publication year	0.03	0.86	(−0.34 to 0.40)
Population (n)	0.00	0.84	(−0.00 to 0.00)
Age	1.31	0.07	(−0.14 to 2.76)
Economic rating	−0.03	0.94	(−1.13 to 1.06)
Weight status	0.96	0.60	(−2.79 to 4.71)
Prevalence of WOB	0.02	0.48	(−0.05 to 0.11)
Status of undernutrition	0.82	0.14	(−0.29 to 1.93)
Prevalence of undernutrition	0.02	0.48	(−0.04 to 0.08)

WOB, Overweight/Obesity.

## Discussion

The aim of this study was to conduct a literature review on the prevalence of DBM in children and adolescents worldwide. The authors included eleven articles that met the inclusion criteria, revealing an individual-level prevalence of DBM of less than 10 %. Despite an increase in knowledge on this topic over the past decade (2013–2022), the authors identified a notable lack of studies addressing DBM in child populations aged 2–19 years, particularly at the individual level in high-income countries compared to low- and middle-income countries.

Among the 11 articles eligible for inclusion in this review, ten were published between 2013 and 2022, suggesting that the topic of individual-level DBM has gained academic interest only in recent years. This growing focus from the scientific community on DBM among children and adolescents can be attributed, at least in part, to the progressive rise in overweight prevalence over the past three decades, affecting increasingly younger age groups.^35^ Consequently, the adverse effects of obesity, such as micronutrient deficiencies and its role as a risk factor for non-communicable diseases (NCDs), have begun to be investigated.^36^ Despite a general decrease in malnutrition prevalence globally, malnutrition issues remain unresolved.^2^^,^^37^ However, there is still a scarcity of studies on individual-level DBM. Comprehensive information on the prevalence of DBM is crucial for investigating and controlling the determinants of this condition, understanding risk distributions, identifying differences between populations or changes within populations over time, and planning preventive measures, treatment, and health service administration.^38^

The prevalence of children and adolescents with a double burden in this review ranged from 1.0 %^22^ to 35.4 %.^24^ This broad variation reflects differences among the various micronutrients examined in conjunction with overweight, the age groups considered, the cut-off points utilized, as well as the influence of different geographic areas, years, and data sources. Furthermore, the heterogeneity in sample sizes and sampling methods inevitably impacts the robustness of the studies. Therefore, studies with small samples and lacking a clear description of sampling methods should be scrutinized, as they may provide weak evidence.

The prevalence of micronutrient deficiency for anemia reached 48.6 %,^20^ while the prevalence of overweight was 37 %^23^ in the studies, with all countries reporting an overweight prevalence exceeding 8 %. This variation in prevalence rates can be partially attributed to biological, social, and environmental factors, including genetics and individual biological differences, rapid changes in dietary patterns (notably the increased consumption of ultra-processed foods and beverages), alterations in physical activity due to urbanization and motorized transport, and cultural differences between countries.^14^ In high-income countries, a low level of malnutrition below 5.5 %^25^^,^^26^^,^^30^ was reported, as expected, since these countries generally exhibit better food conditions, superior social and economic circumstances (such as parental education levels, family housing conditions, and basic sanitation), and improved access to healthcare services.^4^^,^^39^

Currently, research on the DBM has focused on LMIC due to factors such as the nutritional and epidemiological transitions these countries have experienced in recent decades, coinciding with the rising prevalence rates of DBM in their populations.^5^^,^^14^ This finding is consistent with the present study, where the majority of articles (72.7 %) assessed LMIC. Only three^25^^,^^26^^,^^30^ studies from high-income countries were eligible for this review. Although several studies addressing overweight and micronutrient deficiencies involving high-income countries have been reported in the literature, the classification of DBM is relatively new and may not have been utilized in these studies.

Regarding the indicators, the authors observed the use of different cutoff points for overweight (*n* = 5), anemia (*n* = 8), growth retardation (*n* = 2), and different anthropometric standards were used to evaluate deficiencies of different micronutrients (*n* = 5). Some studies used HAZ cutoff values for failure to thrive equal to HAZ < −2 SD or HAZ < 5th percentile as an indicator of malnutrition, while others assessed anemia together or separately with different hemoglobin cutoff points by study and by age, with the most common being anemia (Hb < 120 g/L). However, HAZ better reflects chronic malnutrition,^40^ and the presence of anemia can be caused by the deficiency of several nutrients, so it is preferable that they are used together for studies with children and adolescents on DBM at the individual level.^40^

The classification of excess weight also revealed different indicators, with the most common being BAZ, where a cutoff of BAZ > +1 was utilized. Additionally, BMI was employed with a cutoff point of BMI ≥ 25 for overweight and obesity. However, since BMI varies significantly with advancing age, these cutoff points may not be the most suitable for classifying the nutritional status of children and adolescents. Even among studies using the same z-score, multiple references were applied to assess nutritional status, including international standards from the WHO and the National Center for Health Statistics (NCHS)/WHO.^41^ These varying definitions impact estimates of the prevalence of DBM, and the use of different references can lead to inconsistent classifications of an individual's nutritional status. Therefore, results should be interpreted with caution. The criteria for studies involving DBM appear not to be well established, indicating a significant gap in the literature. Implicit in the varying cutoff points is the potential for non-differential error, which can result in erroneous classification of DBM and an underestimation of exposure.^42^

Among the studies reviewed, only two^27^^,^^30^ (6.22 %) investigated factors associated with DBM, with only one^30^ reporting significant associations. The factors identified as being associated with DBM included ethnicity, parental education, household income, and maternal age. Generally, children from households with lower income and education levels were more likely to experience DBM. Additionally, maternal age was associated with DBM, with children of younger mothers being more vulnerable. The authors suggested that the lack of significant associations in most studies may stem from the complex and multifactorial nature of DBM, necessitating a comprehensive and interdisciplinary approach to its prevention and management.^39^^,^^43^

Three studies^20^^,^^24^^,^^28^ analyzed associations related to excess weight or micronutrient deficiency separately and did not examine the interplay of various factors with the double burden. Two additional articles^21^^,^^23^ explored several factors associated with DBM, frequently assessing urban/rural residence, income, maternal or head-of-household status, and educational attainment in relation to the double burden. However, these studies focused solely on women of reproductive age and did not stratify associations by the age group examined in this review. The limited investigation into factors associated with DBM may be attributed to some studies relying on secondary data, which often provides restricted information. Understanding the relationship between childhood weight status and micronutrient deficiencies, along with the factors influencing this condition, is essential. This underscores the necessity for further analysis targeting the child population at the individual level.

The heterogeneity in the samples and methods used across the analyzed studies likely contributed to the variability in the results, underscoring the need for a more rigorous and standardized approach in collecting and analyzing data related to the Double Burden of Malnutrition (DBM). Such standardization is essential to ensure a more accurate interpretation of findings. These limitations may impact the conclusions of this study, as the lack of standardized terminology can create knowledge gaps and hinder the development of effective interventions. Future research should prioritize using consistent DBM terminology and explore factors associated with DBM in diverse cultural and socioeconomic contexts, which are critical for a comprehensive understanding of this complex issue. Moreover, establishing standardized methods and classification criteria for DBM is imperative for advancing research and guiding public health strategies.

### Potentialities of the study

This is the first systematic review on the topic and represents a significant contribution to scientific knowledge by identifying gaps in the existing literature, particularly the scarcity of investigations focused on this age group. The study highlights a critical area that requires greater attention, such as the standardization of indicators and classification methods to improve comparability between studies, thereby allowing for more robust analyses. This contributes to a deeper understanding of the DBM and its implications for global health.

### Limitations of the study

However, several limitations must be considered, including the possibility of incomplete retrieval of studies. Due to the relatively recent nature of the term "double (or dual) burden of malnutrition," studies that did not adopt this terminology may not have been identified. Furthermore, literature published in languages other than English, Spanish, and Portuguese was not included, even if it provided an abstract written in any of the aforementioned languages.

## Conclusion

The reviewed literature highlights several critical findings: (i) the prevalence of the double burden of malnutrition (DBM) among children and adolescents represents a pressing public health concern, yet research on this condition remains limited; (ii) knowledge of DBM has significantly expanded over the past decade (2013–2022); and (iii) a substantial gap exists in individual-level studies addressing DBM in children and adolescents (ages 2–19) and the associated factors contributing to this condition.

Future research should prioritize establishing standardized definitions and indicators for DBM to enhance data comparability and facilitate more robust analyses. The lack of adolescent-focused studies is particularly concerning, given their vulnerability to micronutrient deficiencies and the influence of unhealthy food marketing, both of which may drive rising obesity rates. Addressing these gaps is essential for advancing the understanding of DBM and informing the development of targeted public health interventions to reduce its burden on young populations.

## Conflicts of interest

The authors declare no conflicts of interest.
